# 6,6′-Di-*tert*-butyl-4,4′-dimethyl-2,2′-[1,2-phenyl­enebis(nitrilo­methanylyl­idene)]diphenol

**DOI:** 10.1107/S1600536811051257

**Published:** 2011-12-07

**Authors:** Rui-Fang Ding, Qi-Bao Wang, Xin-Min Wen

**Affiliations:** a Department of Pharmacy, Jining Medical College, Xueyuan Road 669, Rizhao, People’s Republic of China

## Abstract

In the title mol­ecule, C_30_H_36_N_2_O_2_, the dihedral angles between the central benzene ring and the two benzene rings of the butyl­salicylaldimine groups are 14.3 (2) and 40.6 (2)°. There are two strong intra­molecular O—H⋯N hydrogen bonds which form *S*(6) rings. The crystal studied was a non-merohedral twin with refined components of 0.270 (4) and 0.730 (4).

## Related literature

For applications of Schiff base ligands in pharmaceutical and catalytic research, see: Hashimoto & Maruoka (2007 ▶); Singh *et al.* (2009 ▶). For a related structure, see: You *et al.* (2010 ▶). For hydrogen-bond motifs, see: Bernstein *et al.* (1995 ▶).
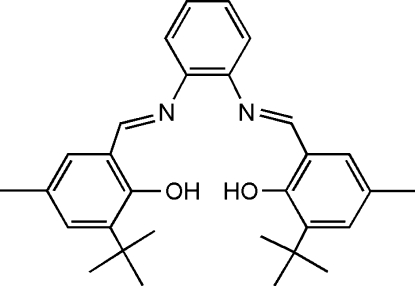


## Experimental

### 

#### Crystal data

C_30_H_36_N_2_O_2_
*M* *_r_* = 456.61
Triclinic, 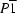

*a* = 10.578 (7) Å
*b* = 11.394 (7) Å
*c* = 12.217 (7) Å
α = 72.195 (6)°
β = 73.525 (6)°
γ = 72.975 (6)°
*V* = 1309.8 (14) Å^3^
*Z* = 2
Mo *K*α radiation
μ = 0.07 mm^−1^
*T* = 296 K
0.28 × 0.22 × 0.15 mm

#### Data collection

Bruker SMART CCD diffractometer
Absorption correction: multi-scan (*SADABS*; Bruker, 2007 ▶) *T*_min_ = 0.980, *T*_max_ = 0.989
4593 measured reflections
4593 independent reflections
2894 reflections with *I* > 2σ(*I*)

#### Refinement

*R*[*F* ^2^ > 2σ(*F* ^2^)] = 0.075
*wR*(*F* ^2^) = 0.271
*S* = 1.13
4593 reflections
318 parameters
H-atom parameters constrained
Δρ_max_ = 0.29 e Å^−3^
Δρ_min_ = −0.30 e Å^−3^

### 

Data collection: *SMART* (Bruker, 2007 ▶); cell refinement: *SAINT* (Bruker, 2007 ▶); data reduction: *SAINT*; program(s) used to solve structure: *SHELXS97* (Sheldrick, 2008 ▶); program(s) used to refine structure: *SHELXL97* (Sheldrick, 2008 ▶); molecular graphics: *SHELXTL* (Sheldrick, 2008 ▶) and *DIAMOND* (Brandenburg, 1999 ▶); software used to prepare material for publication: *SHELXTL* and *PLATON* (Spek, 2009 ▶).

## Supplementary Material

Crystal structure: contains datablock(s) I, global. DOI: 10.1107/S1600536811051257/lh5371sup1.cif

Structure factors: contains datablock(s) I. DOI: 10.1107/S1600536811051257/lh5371Isup2.hkl

Additional supplementary materials:  crystallographic information; 3D view; checkCIF report

## Figures and Tables

**Table 1:** Hydrogen-bond geometry (Å, °)

*D*—H⋯*A*	*D*—H	H⋯*A*	*D*⋯*A*	*D*—H⋯*A*
O1—H1⋯N1	0.82	1.89	2.605 (4)	145
O2—H2⋯N2	0.82	1.87	2.609 (4)	149

**Fig. 1.**
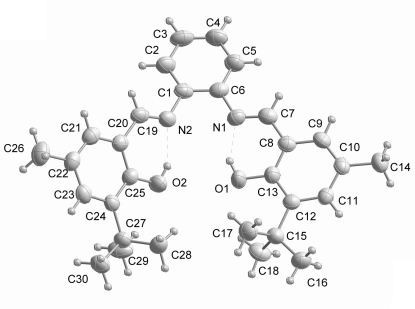
The molecular structure of title compound. Displacement ellipsoids are drawn at the 50% probability level.

## supplementary crystallographic information

### Comment

Schiff base ligands attract much attention in pharmaceutical fields as well as in catalytic research. Here we report the molecular structure of a tetradentate Schiff base ligand, which is shown in Fig. 1.

The dihedral angles between the central benzene ring and the two benzene rings butylsalicylaldimine groups are 14.3 (2)° (C8-C13) and 40.6 (2)° (C20-C25). There are two strong intramolecular O—H···N hydrogen bonds which form S(6) rings (Bernstein et al., 1995). The hydrogen bonding in the title compound is different to that reported in the related structure (You et al., 2010) possibly owing to the steric effects of the bulky t-butyl substituents.

### Experimental

5-methyl-3-*t*-butyl-2-hydroxybenzaldehyde (0.192 g, 1 mmol) dissolved in 20 ml ethanol, then 1,2-phenylenediamine (0.043 g, 0.5 mmol) in 20 ml ethanol was added. The mixture was stirred at 323K for 5 h. The solution was cooled to room temperature and the resulting orange solid was collected, washed by cold ethanol and dried *in vacuo*. Yield: 0.175 g, 80.6%. Cooling the ethanol solution to room temperature gave orange crystals suitable for X-ray diffraction measurement.

### Refinement

All H atoms were positioned geometrically and refined using a riding model with C—H = 0.93–0.97 Å; O—H = 0.82Å and with *U*_iso_(H) = 1.2 times *U*_eq_(C) or *U*_iso_(H) = 1.5 times *U*_eq_(C_methyl_,O). Analysis of the structure in PLATON (Spek, 2009) revealed the crystal was a non-merohedral twin with twin law (110)[432]. The ratio of the twin components refined to 0.270 (4):0.730 (4).

### Crystal data

**Table:**

C_30_H_36_N_2_O_2_	*Z* = 2
*M**_r_* = 456.61	*F*(000) = 492
Triclinic, *P*1	*D*_x_ = 1.158 Mg m^−^^3^
Hall symbol: -P 1	Mo *K*α radiation, λ = 0.71073 Å
*a* = 10.578 (7) Å	Cell parameters from 3022 reflections
*b* = 11.394 (7) Å	θ = 2.4–24.5°
*c* = 12.217 (7) Å	µ = 0.07 mm^−^^1^
α = 72.195 (6)°	*T* = 296 K
β = 73.525 (6)°	Block, orange
γ = 72.975 (6)°	0.28 × 0.22 × 0.15 mm
*V* = 1309.8 (14) Å^3^	

### Data collection

**Table:**

Bruker SMART CCD diffractometer	4593 independent reflections
Radiation source: fine-focus sealed tube	2894 reflections with *I* > 2σ(*I*)
graphite	*R*_int_ = 0.000
φ and ω scans	θ_max_ = 25.0°, θ_min_ = 2.4°
Absorption correction: multi-scan (*SADABS*; Bruker, 2007)	*h* = −12→12
*T*_min_ = 0.980, *T*_max_ = 0.989	*k* = −13→13
4593 measured reflections	*l* = −13→14

### Refinement

**Table:**

Refinement on *F*^2^	Primary atom site location: structure-invariant direct methods
Least-squares matrix: full	Secondary atom site location: difference Fourier map
*R*[*F*^2^ > 2σ(*F*^2^)] = 0.075	Hydrogen site location: inferred from neighbouring sites
*wR*(*F*^2^) = 0.271	H-atom parameters constrained
*S* = 1.13	*w* = 1/[σ^2^(*F*_o_^2^) + (0.1477*P*)^2^ + 0.0358*P*] where *P* = (*F*_o_^2^ + 2*F*_c_^2^)/3
4593 reflections	(Δ/σ)_max_ = 0.001
318 parameters	Δρ_max_ = 0.29 e Å^−^^3^
0 restraints	Δρ_min_ = −0.30 e Å^−^^3^

### Special details

**Table:**

Geometry. All e.s.d.'s (except the e.s.d. in the dihedral angle between two l.s. planes) are estimated using the full covariance matrix. The cell e.s.d.'s are taken into account individually in the estimation of e.s.d.'s in distances, angles and torsion angles; correlations between e.s.d.'s in cell parameters are only used when they are defined by crystal symmetry. An approximate (isotropic) treatment of cell e.s.d.'s is used for estimating e.s.d.'s involving l.s. planes.
Refinement. Refinement of *F*^2^ against ALL reflections. The weighted *R*-factor *wR* and goodness of fit *S* are based on *F*^2^, conventional *R*-factors *R* are based on *F*, with *F* set to zero for negative *F*^2^. The threshold expression of *F*^2^ > σ(*F*^2^) is used only for calculating *R*-factors(gt) *etc*. and is not relevant to the choice of reflections for refinement. *R*-factors based on *F*^2^ are statistically about twice as large as those based on *F*, and *R*- factors based on ALL data will be even larger.

### Fractional atomic coordinates and isotropic or equivalent isotropic displacement parameters (Å^2^)

**Table:**

	*x*	*y*	*z*	*U*_iso_*/*U*_eq_	
N1	0.2830 (3)	0.5924 (3)	0.9145 (3)	0.0503 (7)	
N2	0.4261 (3)	0.7498 (3)	0.7380 (2)	0.0519 (8)	
O1	0.0609 (2)	0.7680 (2)	0.9079 (2)	0.0631 (8)	
H1	0.1431	0.7403	0.8950	0.095*	
O2	0.2253 (2)	0.9257 (2)	0.6653 (3)	0.0643 (8)	
H2	0.2684	0.8546	0.6911	0.096*	
C1	0.4186 (3)	0.5407 (3)	0.8651 (3)	0.0481 (9)	
C2	0.4834 (4)	0.4144 (4)	0.8975 (4)	0.0618 (10)	
H2A	0.4367	0.3594	0.9571	0.074*	
C3	0.6136 (4)	0.3682 (4)	0.8448 (4)	0.0673 (11)	
H3	0.6542	0.2829	0.8682	0.081*	
C4	0.6842 (4)	0.4480 (4)	0.7573 (4)	0.0667 (11)	
H4	0.7729	0.4171	0.7213	0.080*	
C5	0.6236 (4)	0.5733 (4)	0.7231 (3)	0.0616 (10)	
H5	0.6717	0.6269	0.6632	0.074*	
C6	0.4910 (3)	0.6220 (3)	0.7765 (3)	0.0494 (9)	
C7	0.2184 (3)	0.5328 (3)	1.0093 (3)	0.0513 (9)	
H7	0.2646	0.4556	1.0486	0.062*	
C8	0.0780 (3)	0.5772 (3)	1.0597 (3)	0.0474 (8)	
C9	0.0143 (4)	0.5004 (3)	1.1613 (3)	0.0507 (9)	
H9	0.0650	0.4232	1.1960	0.061*	
C10	−0.1199 (3)	0.5353 (3)	1.2109 (3)	0.0479 (8)	
C11	−0.1908 (3)	0.6540 (3)	1.1575 (3)	0.0462 (8)	
H11	−0.2815	0.6801	1.1916	0.055*	
C12	−0.1352 (3)	0.7354 (3)	1.0573 (3)	0.0443 (8)	
C13	0.0023 (3)	0.6937 (3)	1.0077 (3)	0.0473 (8)	
C14	−0.1909 (4)	0.4504 (4)	1.3160 (3)	0.0625 (10)	
H14A	−0.1396	0.3646	1.3231	0.094*	
H14B	−0.2794	0.4561	1.3060	0.094*	
H14C	−0.1988	0.4763	1.3858	0.094*	
C15	−0.2195 (3)	0.8652 (3)	1.0034 (3)	0.0493 (9)	
C16	−0.3626 (4)	0.8925 (4)	1.0780 (4)	0.0667 (11)	
H16A	−0.3579	0.8885	1.1564	0.100*	
H16B	−0.4093	0.8308	1.0805	0.100*	
H16C	−0.4105	0.9754	1.0440	0.100*	
C17	−0.1554 (4)	0.9714 (4)	0.9985 (4)	0.0663 (11)	
H17A	−0.2100	1.0516	0.9665	0.099*	
H17B	−0.0662	0.9608	0.9494	0.099*	
H17C	−0.1499	0.9684	1.0764	0.099*	
C18	−0.2286 (4)	0.8684 (4)	0.8795 (3)	0.0718 (12)	
H18A	−0.2766	0.9509	0.8444	0.108*	
H18B	−0.2759	0.8062	0.8843	0.108*	
H18C	−0.1391	0.8502	0.8322	0.108*	
C19	0.4916 (3)	0.8371 (4)	0.7072 (3)	0.0520 (9)	
H19	0.5806	0.8146	0.7161	0.062*	
C20	0.4349 (3)	0.9685 (3)	0.6595 (3)	0.0473 (8)	
C21	0.5145 (3)	1.0562 (4)	0.6317 (3)	0.0538 (9)	
H21	0.6034	1.0280	0.6413	0.065*	
C22	0.4643 (3)	1.1837 (4)	0.5903 (3)	0.0515 (9)	
C23	0.3297 (3)	1.2211 (3)	0.5787 (3)	0.0509 (9)	
H23	0.2936	1.3072	0.5537	0.061*	
C24	0.2463 (3)	1.1391 (3)	0.6016 (3)	0.0459 (8)	
C25	0.3013 (3)	1.0107 (3)	0.6425 (3)	0.0471 (8)	
C26	0.5487 (4)	1.2799 (4)	0.5618 (4)	0.0772 (12)	
H26A	0.5223	1.3229	0.6240	0.116*	
H26B	0.5349	1.3402	0.4892	0.116*	
H26C	0.6426	1.2377	0.5541	0.116*	
C27	0.1000 (3)	1.1870 (3)	0.5839 (3)	0.0521 (9)	
C28	−0.0004 (4)	1.1451 (4)	0.6987 (4)	0.0799 (14)	
H28A	−0.0906	1.1728	0.6849	0.120*	
H28B	0.0051	1.1817	0.7578	0.120*	
H28C	0.0216	1.0545	0.7251	0.120*	
C29	0.0872 (4)	1.1343 (4)	0.4862 (4)	0.0758 (13)	
H29A	0.1115	1.0435	0.5082	0.114*	
H29B	0.1466	1.1647	0.4139	0.114*	
H29C	−0.0044	1.1618	0.4759	0.114*	
C30	0.0582 (4)	1.3314 (4)	0.5466 (4)	0.0647 (11)	
H30A	−0.0328	1.3563	0.5348	0.097*	
H30B	0.1181	1.3618	0.4746	0.097*	
H30C	0.0631	1.3666	0.6069	0.097*	

### Atomic displacement parameters (Å^2^)

**Table:**

	*U*^11^	*U*^22^	*U*^33^	*U*^12^	*U*^13^	*U*^23^
N1	0.0436 (15)	0.0505 (18)	0.0563 (18)	−0.0057 (13)	−0.0101 (14)	−0.0173 (15)
N2	0.0491 (16)	0.0522 (19)	0.0444 (16)	−0.0018 (15)	−0.0067 (13)	−0.0099 (14)
O1	0.0528 (14)	0.0578 (17)	0.0587 (16)	−0.0115 (12)	0.0020 (12)	0.0004 (13)
O2	0.0534 (15)	0.0452 (15)	0.087 (2)	−0.0099 (12)	−0.0198 (14)	−0.0028 (14)
C1	0.0416 (18)	0.051 (2)	0.054 (2)	−0.0017 (16)	−0.0157 (16)	−0.0193 (17)
C2	0.059 (2)	0.048 (2)	0.074 (3)	−0.0061 (18)	−0.0136 (19)	−0.0160 (19)
C3	0.062 (2)	0.051 (2)	0.085 (3)	0.006 (2)	−0.024 (2)	−0.022 (2)
C4	0.048 (2)	0.070 (3)	0.073 (3)	0.011 (2)	−0.0123 (19)	−0.029 (2)
C5	0.050 (2)	0.066 (3)	0.055 (2)	0.0009 (19)	−0.0050 (17)	−0.0155 (19)
C6	0.0471 (19)	0.054 (2)	0.0427 (19)	0.0037 (16)	−0.0139 (15)	−0.0156 (16)
C7	0.051 (2)	0.050 (2)	0.054 (2)	−0.0072 (17)	−0.0147 (17)	−0.0160 (17)
C8	0.0481 (19)	0.050 (2)	0.0460 (19)	−0.0087 (16)	−0.0132 (15)	−0.0144 (16)
C9	0.057 (2)	0.047 (2)	0.048 (2)	−0.0074 (16)	−0.0198 (16)	−0.0065 (16)
C10	0.055 (2)	0.051 (2)	0.0390 (17)	−0.0168 (17)	−0.0150 (15)	−0.0042 (15)
C11	0.0444 (18)	0.055 (2)	0.0429 (19)	−0.0156 (16)	−0.0097 (14)	−0.0127 (16)
C12	0.0442 (18)	0.048 (2)	0.0417 (18)	−0.0101 (15)	−0.0112 (14)	−0.0108 (15)
C13	0.0501 (19)	0.050 (2)	0.0397 (17)	−0.0143 (16)	−0.0068 (15)	−0.0072 (15)
C14	0.068 (2)	0.064 (3)	0.052 (2)	−0.025 (2)	−0.0168 (18)	0.0032 (18)
C15	0.0489 (19)	0.049 (2)	0.0466 (19)	−0.0091 (16)	−0.0141 (15)	−0.0057 (16)
C16	0.053 (2)	0.062 (3)	0.075 (3)	−0.0029 (19)	−0.0141 (19)	−0.012 (2)
C17	0.068 (2)	0.047 (2)	0.084 (3)	−0.0155 (19)	−0.020 (2)	−0.010 (2)
C18	0.083 (3)	0.073 (3)	0.058 (2)	−0.005 (2)	−0.031 (2)	−0.011 (2)
C19	0.0457 (19)	0.057 (2)	0.0437 (19)	−0.0007 (18)	−0.0059 (15)	−0.0124 (17)
C20	0.0424 (18)	0.055 (2)	0.0398 (17)	−0.0069 (16)	−0.0041 (14)	−0.0138 (15)
C21	0.0453 (19)	0.068 (3)	0.049 (2)	−0.0123 (18)	−0.0056 (16)	−0.0206 (18)
C22	0.053 (2)	0.058 (2)	0.0451 (19)	−0.0205 (18)	−0.0026 (16)	−0.0152 (17)
C23	0.059 (2)	0.047 (2)	0.0417 (18)	−0.0128 (17)	−0.0026 (16)	−0.0112 (15)
C24	0.0469 (18)	0.047 (2)	0.0397 (18)	−0.0097 (16)	−0.0056 (14)	−0.0086 (15)
C25	0.0437 (18)	0.051 (2)	0.0442 (18)	−0.0128 (16)	−0.0042 (14)	−0.0111 (15)
C26	0.078 (3)	0.083 (3)	0.081 (3)	−0.040 (2)	−0.009 (2)	−0.021 (2)
C27	0.0487 (19)	0.045 (2)	0.055 (2)	−0.0066 (16)	−0.0099 (16)	−0.0072 (16)
C28	0.049 (2)	0.074 (3)	0.086 (3)	−0.008 (2)	−0.001 (2)	0.007 (2)
C29	0.072 (3)	0.068 (3)	0.100 (3)	−0.007 (2)	−0.038 (2)	−0.027 (2)
C30	0.062 (2)	0.049 (2)	0.073 (3)	−0.0042 (18)	−0.016 (2)	−0.0084 (19)

### Geometric parameters (Å, °)

**Table:**

N1—C7	1.270 (4)	C16—H16A	0.9600
N1—C1	1.414 (4)	C16—H16B	0.9600
N2—C19	1.280 (5)	C16—H16C	0.9600
N2—C6	1.411 (4)	C17—H17A	0.9600
O1—C13	1.354 (4)	C17—H17B	0.9600
O1—H1	0.8200	C17—H17C	0.9600
O2—C25	1.351 (4)	C18—H18A	0.9600
O2—H2	0.8200	C18—H18B	0.9600
C1—C2	1.387 (5)	C18—H18C	0.9600
C1—C6	1.396 (5)	C19—C20	1.441 (5)
C2—C3	1.368 (5)	C19—H19	0.9300
C2—H2A	0.9300	C20—C21	1.395 (5)
C3—C4	1.370 (6)	C20—C25	1.407 (5)
C3—H3	0.9300	C21—C22	1.378 (5)
C4—C5	1.370 (6)	C21—H21	0.9300
C4—H4	0.9300	C22—C23	1.397 (5)
C5—C6	1.395 (5)	C22—C26	1.511 (5)
C5—H5	0.9300	C23—C24	1.382 (5)
C7—C8	1.440 (5)	C23—H23	0.9300
C7—H7	0.9300	C24—C25	1.396 (5)
C8—C13	1.399 (5)	C24—C27	1.536 (5)
C8—C9	1.403 (5)	C26—H26A	0.9600
C9—C10	1.367 (5)	C26—H26B	0.9600
C9—H9	0.9300	C26—H26C	0.9600
C10—C11	1.401 (5)	C27—C30	1.529 (5)
C10—C14	1.505 (5)	C27—C28	1.538 (5)
C11—C12	1.385 (5)	C27—C29	1.544 (6)
C11—H11	0.9300	C28—H28A	0.9600
C12—C13	1.407 (5)	C28—H28B	0.9600
C12—C15	1.543 (5)	C28—H28C	0.9600
C14—H14A	0.9600	C29—H29A	0.9600
C14—H14B	0.9600	C29—H29B	0.9600
C14—H14C	0.9600	C29—H29C	0.9600
C15—C16	1.528 (5)	C30—H30A	0.9600
C15—C17	1.530 (5)	C30—H30B	0.9600
C15—C18	1.533 (5)	C30—H30C	0.9600
			
C7—N1—C1	121.6 (3)	C15—C17—H17B	109.5
C19—N2—C6	120.5 (3)	H17A—C17—H17B	109.5
C13—O1—H1	109.5	C15—C17—H17C	109.5
C25—O2—H2	109.5	H17A—C17—H17C	109.5
C2—C1—C6	118.0 (3)	H17B—C17—H17C	109.5
C2—C1—N1	124.4 (3)	C15—C18—H18A	109.5
C6—C1—N1	117.6 (3)	C15—C18—H18B	109.5
C3—C2—C1	122.1 (4)	H18A—C18—H18B	109.5
C3—C2—H2A	118.9	C15—C18—H18C	109.5
C1—C2—H2A	118.9	H18A—C18—H18C	109.5
C2—C3—C4	119.7 (4)	H18B—C18—H18C	109.5
C2—C3—H3	120.1	N2—C19—C20	123.7 (3)
C4—C3—H3	120.1	N2—C19—H19	118.2
C3—C4—C5	119.7 (4)	C20—C19—H19	118.2
C3—C4—H4	120.1	C21—C20—C25	119.3 (3)
C5—C4—H4	120.1	C21—C20—C19	118.8 (3)
C4—C5—C6	121.2 (4)	C25—C20—C19	121.9 (3)
C4—C5—H5	119.4	C22—C21—C20	121.6 (3)
C6—C5—H5	119.4	C22—C21—H21	119.2
C5—C6—C1	119.2 (3)	C20—C21—H21	119.2
C5—C6—N2	121.4 (3)	C21—C22—C23	116.9 (3)
C1—C6—N2	119.4 (3)	C21—C22—C26	122.0 (4)
N1—C7—C8	124.0 (3)	C23—C22—C26	121.1 (3)
N1—C7—H7	118.0	C24—C23—C22	124.6 (3)
C8—C7—H7	118.0	C24—C23—H23	117.7
C13—C8—C9	119.1 (3)	C22—C23—H23	117.7
C13—C8—C7	121.5 (3)	C23—C24—C25	116.6 (3)
C9—C8—C7	119.3 (3)	C23—C24—C27	121.7 (3)
C10—C9—C8	122.1 (3)	C25—C24—C27	121.7 (3)
C10—C9—H9	119.0	O2—C25—C24	119.5 (3)
C8—C9—H9	119.0	O2—C25—C20	119.6 (3)
C9—C10—C11	116.8 (3)	C24—C25—C20	121.0 (3)
C9—C10—C14	122.4 (3)	C22—C26—H26A	109.5
C11—C10—C14	120.8 (3)	C22—C26—H26B	109.5
C12—C11—C10	124.6 (3)	H26A—C26—H26B	109.5
C12—C11—H11	117.7	C22—C26—H26C	109.5
C10—C11—H11	117.7	H26A—C26—H26C	109.5
C11—C12—C13	116.5 (3)	H26B—C26—H26C	109.5
C11—C12—C15	121.7 (3)	C30—C27—C24	112.7 (3)
C13—C12—C15	121.7 (3)	C30—C27—C28	106.6 (3)
O1—C13—C8	120.1 (3)	C24—C27—C28	110.9 (3)
O1—C13—C12	119.0 (3)	C30—C27—C29	107.8 (3)
C8—C13—C12	120.9 (3)	C24—C27—C29	108.7 (3)
C10—C14—H14A	109.5	C28—C27—C29	110.1 (4)
C10—C14—H14B	109.5	C27—C28—H28A	109.5
H14A—C14—H14B	109.5	C27—C28—H28B	109.5
C10—C14—H14C	109.5	H28A—C28—H28B	109.5
H14A—C14—H14C	109.5	C27—C28—H28C	109.5
H14B—C14—H14C	109.5	H28A—C28—H28C	109.5
C16—C15—C17	106.0 (3)	H28B—C28—H28C	109.5
C16—C15—C18	108.8 (3)	C27—C29—H29A	109.5
C17—C15—C18	110.1 (3)	C27—C29—H29B	109.5
C16—C15—C12	111.8 (3)	H29A—C29—H29B	109.5
C17—C15—C12	110.3 (3)	C27—C29—H29C	109.5
C18—C15—C12	109.7 (3)	H29A—C29—H29C	109.5
C15—C16—H16A	109.5	H29B—C29—H29C	109.5
C15—C16—H16B	109.5	C27—C30—H30A	109.5
H16A—C16—H16B	109.5	C27—C30—H30B	109.5
C15—C16—H16C	109.5	H30A—C30—H30B	109.5
H16A—C16—H16C	109.5	C27—C30—H30C	109.5
H16B—C16—H16C	109.5	H30A—C30—H30C	109.5
C15—C17—H17A	109.5	H30B—C30—H30C	109.5

### Hydrogen-bond geometry (Å, °)

**Table:**

*D*—H···*A*	*D*—H	H···*A*	*D*···*A*	*D*—H···*A*
O1—H1···N1	0.82	1.89	2.605 (4)	145.
O2—H2···N2	0.82	1.87	2.609 (4)	149.
